# Vacuum level dependent photoluminescence in chemical vapor deposition-grown monolayer MoS_**2**_

**DOI:** 10.1038/s41598-017-15577-1

**Published:** 2017-12-01

**Authors:** Linfeng Sun, Xiaoming Zhang, Fucai Liu, Youde Shen, Xiaofeng Fan, Shoujun Zheng, John T. L. Thong, Zheng Liu, Shengyuan A. Yang, Hui Ying Yang

**Affiliations:** 1 Pillar of Engineering Product Development, Singapore University of Technology and Design, Singapore, 487372 Singapore; 2 Division of Physics and Applied Physics, School of Physical and Mathematical Science, Nanyang Technological University, Singapore, 637371 Singapore; 3 Center for Programmable Materials, School of Materials Science and Engineering, Nanyang Technological University, Singapore, 639798 Singapore; 4 Department of Electrical and Computer Engineering, National University of Singapore, Singapore, 117583 Singapore; 5 College of Materials Science and Engineering, Jilin University, Changchun, 130012 P. R. China; 6 Centre for Micro-/Nano-electronics (NOVITAS), School of Electrical & Electronic Engineering, Nanyang Technological University, Singapore, 639798 Singapore

## Abstract

The stronger photoluminescence (PL) in chemical vapor deposition (CVD) grown monolayer MoS_2_ has been attributed to its high crystal quality compared with that in mechanically exfoliated (ME) crystal, which is contrary to the cognition that the ME crystal usually have better crystal quality than that of CVD grown one and it is expected with a better optical quality. In this report, the reason of abnormally strong PL spectra in CVD grown monolayer crystal is systematically investigated by studying the *in-situ* opto-electrical exploration at various environments for both of CVD and ME samples. High resolution transmission electron microscopy is used to investigate their crystal qualities. The stronger PL in CVD grown crystal is due to the high p-doping effect of adsorbates induced rebalance of exciton/trion emission. The first principle calculations are carried out to explore the interaction between adsorbates in ambient and defects sites in MoS_2_, which is consistent to the experimental phenomenon and further confirm our proposed mechanisms.

## Introduction

Transition metal dichalcogenides (TMDs) have attracted tremendous attention due to their extraordinary optoelectrical properties, setting the stage for new breakthroughs in materials science, especially when the thickness of these materials approaches atomic-level thicknesses^1–7^. Molybdenum disulfide (MoS_2_), as a typical TMD, has experimentally demonstrated high mobility and high on/off ratio when used as an active material in electronics devices, while the circularly polarized photoluminescence (PL) for single layer MoS_2_ makes it promising for valleytronic devices^7–10^. Though single layer MoS_2_ is a good candidate for its application in electronic devices, the size of samples fabricated by ME method limits its practical use. Therefore, CVD method has been used to synthesize large-area samples although the electrical properties of CVD grown samples are poorer than that of ME samples^11–15^. However, CVD grown monolayer MoS_2_ displays abnormally optical quality with a stronger PL intensity compared to that of ME samples, which has been observed in previous reports and attributed to the proof of high crystal quality^16,17^, since optical performance at room temperature is usually used to evaluate the crystal quality of two dimensional (2D) semiconductors, based on the premise that intense PL emission is observed for low-defective compound semiconductors^18^. However, this conclusion cannot work here, because the crystal quality of ME samples is usually better than that of CVD grown samples and is expected with a better optical quality. Till now, it is still a controversial issue that the precious explanation for the stronger PL in CVD grown samples than that of ME sample is due to its higher crystal quality^16,17,19–23^. Thus, the mechanism for the strong PL in CVD grown samples is still lacking and the clarification for this doubt in this work will be desirable and helpful to recognize its nature of stronger PL in CVD grown samples. In this work, by investigating the optical spectra of monolayer MoS_2_ samples with different amounts of defects, as well as their electrical performance, we uncover the emission mechanism of abnormally strong PL observed in CVD grown samples and attribute it to the p doping effect from the larger amounts of adsorbates on the defect sites of MoS_2_, which rebalance the radiative emission intensities of excitons.

## Results and Discussion

**Figure 1:**
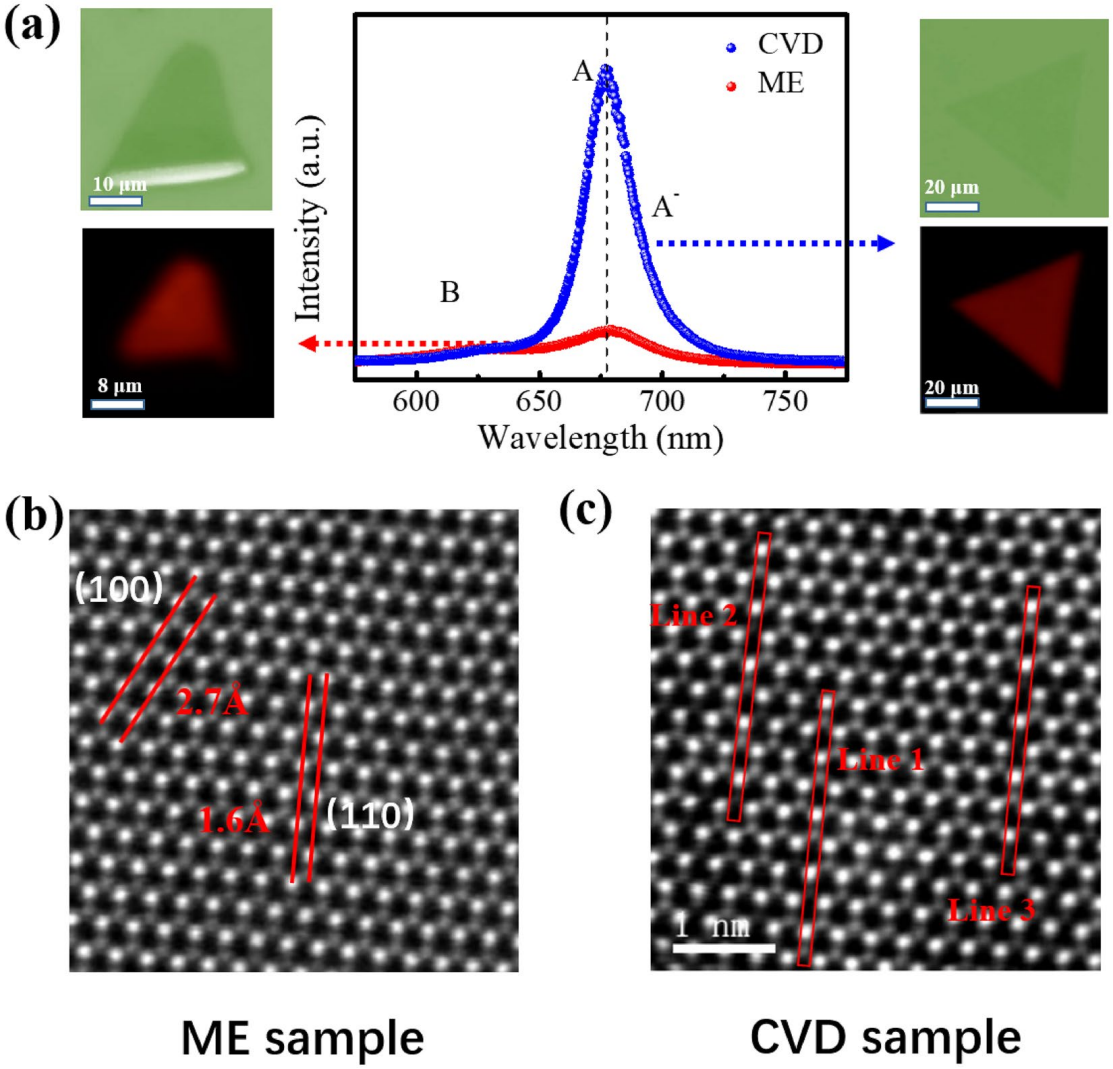
(**a**) PL spectra of single layer ME and CVD grown MoS_2_. Left column: optical image of a typical monolayer sample fabricated by ME method and its PL mapping. Right column: optical image of a typical CVD-grown monolayer sample and its PL mapping. Both of two PL mappings are normalized to the intensity of A exciton. (**b**) and (**c**) are the HAADF image of ME and CVD samples, respectively. The lines indicate the defects observed in this image.

Figure 1a shows the PL spectra of typical CVD grown and ME monolayer MoS_2_. Their corresponding PL mappings show the uniform intensities. Clearly, the PL intensity of the former is stronger than that of the latter, which was previously attributed to the high crystal quality of CVD grown crystal^16,17^. The X-ray photoelectron spectroscopies for these two groups of samples are carried out to show the atomic ratio (S/Mo), as shown in Fig. S1. For CVD grown sample, the peaks at 162.3 and 163.4 eV are assigned to the S^2−^ 2p_3/2_ and 2p_1/2_, respectively. The peaks at 229.5 and 232.7 eV are attributed to the Mo^4+^ 3d_5/2_ and 3d_3/2_. While for ME sample, the peaks at 162.6 and 163.6 eV are assigned to the 2p3/2 and 2p1/2, of divalent sulphide ions (S^2−^) and the peaks at 229.67 and 232.8 eV are from the 3d_5/2_ and 3d_3/2_ of the core levels of Mo^4+^. The S/Mo ratio for CVD sample is 1.66 and that for ME sample is 1.86, which is consistent to the results of EDS and more defects are introduced in CVD grown samples. Figure 1b,c show typical high resolution atomic structure generated by high angle annular dark field (HAADF) scanning transmission electron microscopy (STEM), and they clearly display the defects in CVD grown monolayer MoS_2_, as indicated in the lines. The lattice spaces of hexagonal lattice structure for MoS_2_ is 2.7 Å ((100) plane) and 1.6 Å ((110) plane). The corresponding intensity line profiles shows in Fig. S2 display most of defects are single S vacancy. Fig. S3a gives a directly visual sense of the effect of defects on the PL intensities of ME MoS_2_. The marked circle area with pre-exposed by Argon (Ar) plasma shows stronger PL intensity. Fig. S3b shows the PL spectra of area A (exposed by Ar plasma) and B (without Ar plasma treatment), respectively. Therefore, it seems that the more defects contribute to the stronger PL emission in MoS_2_. Nevertheless, whether the PL spectra observed in MoS_2_ is its intrinsic emission or defect-induced emission remains unclear. The previous reports have revealed the energy of defect-induced PL is around 1.78 eV, at the low energy side of the reported A exciton^22^. However, as shown in Fig. 1, there is no extra emission peak except a broadened shoulder, which has been identified as the trion emission, marked as A^−^ exciton emission. Moreover, the temperature dependent PL spectra (shown in Fig. S4) indicates the PL peak observed is an intrinsic emission, and the match among the electron spin, the layer pseudospin and the valley pseudospin reported in the literature display the features of intrinsic emission^6–9,24^. Thus the observed PL peak in Fig. 1 is not the defect-induced emission in MoS_2_. Another potential factor that may lead to the difference of optical properties between CVD grown and ME samples is the strain effect from the substrate since the contact behaviours between the substrate and the sample are different for these two cases. The CVD grown samples are inevitably affected by the strain effect because of the different thermal expansion coefficients between deposited layer and substrate during the cooling process^25–27^. Figure S5 compares the Raman spectra of both of CVD grown and ME sample. The ${{\rm{E}}}_{2{\rm{g}}}^{1}$ and A_1g_ modes of CVD grown samples show a red-shift and blue-shift, respectively, when compared with those of ME 1 L MoS_2_ sample. This phenomenon differs from the variation trend when strain is applied^28^. Also, if the strain effect is the main factor in the PL spectra of MoS_2_, the energies of PL would shift clearly towards higher energy (compressive strain) and lower energy (tensile strain), respectively^29,30^, but there is no obvious shift of peak position shown in Fig. 1. Thus, the strain effect could be excluded in this study. So far, defects are most likely to be responsible for the enhancement of photon emission in CVD grown monolayer MoS_2_.

**Figure 2:**
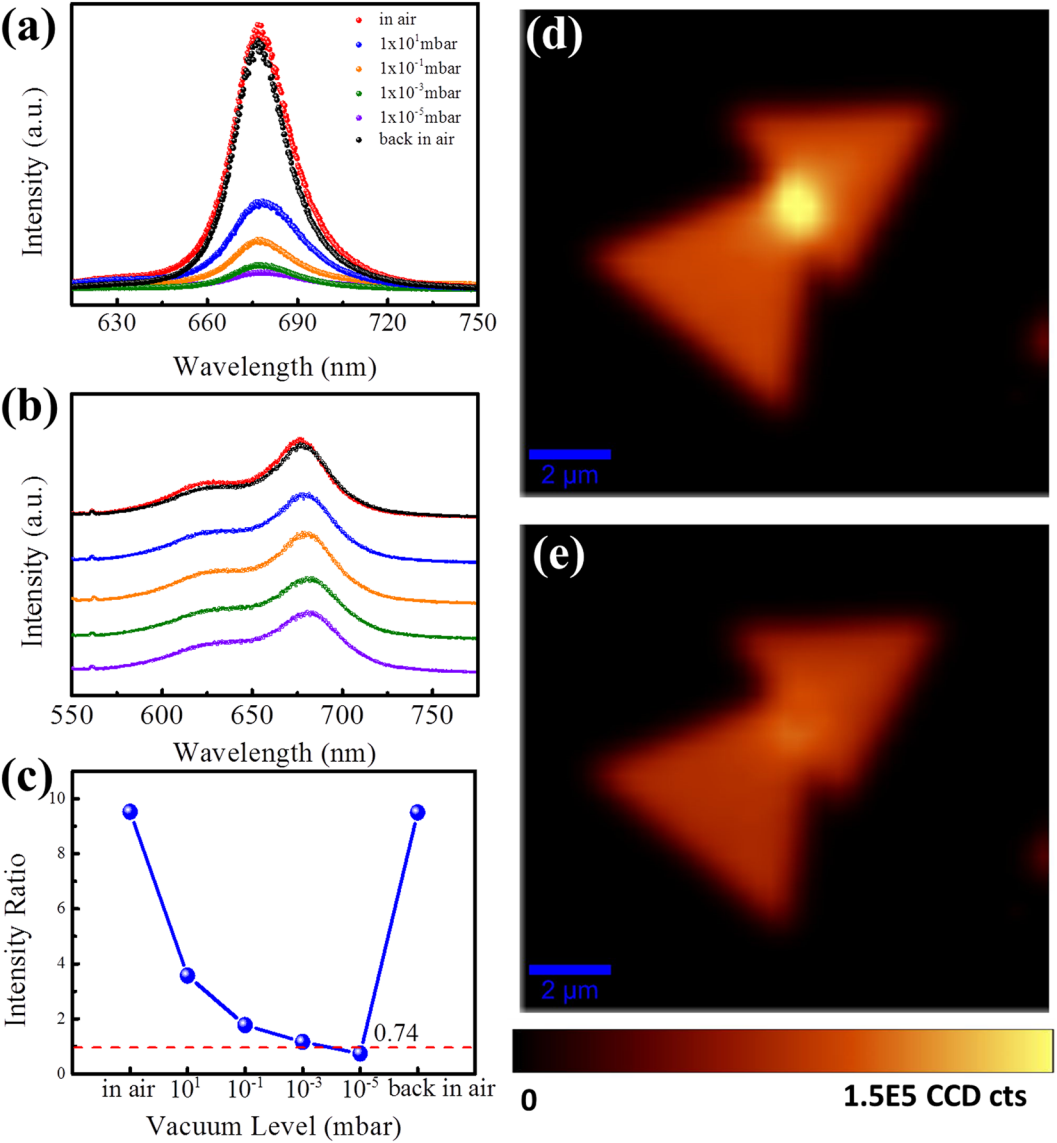
Vacuum level dependent PL spectra of (**a**) CVD grown and (**b**) ME MoS_2_ samples. (**c**) The ratios of PL intensities between CVD grown and ME MoS_2_ samples at different vacuum levels shown in Figure (**a**,**b**). The PL mappings of a typical CVD-grown monolayer MoS_2_ measured in air (**d**) and vacuum (**e**), respectively. Both of PL mappings are normalized to the intensity of A exciton.

Since defects in atomically layered materials usually could act as adsorption centres due to their higher active adsorption energy, the PL spectra of CVD and ME MoS_2_ sample were measured in vacuum to exclude the role of adsorbates. Surprisingly, as shown in Fig. 2a, the PL intensity of CVD grown MoS_2_ decreased dramatically in vacuum. At the same time, the emission intensities are reversible when the CVD grown samples exposed in air again. However, for the PL spectra of ME sample (Fig. 2b), its intensity does not show obvious variations with the ambient varies. The reduction of PL intensity in vacuum for CVD grown sample implies that the adsorbates adsorbed on the surface of MoS_2_ greatly affect its photon emission. According to the vacuum-level dependent PL intensities, it seems that more adsorbates contribute to stronger PL intensity. Moreover, it is found that in Fig. 2c, the ratio of PL intensities between CVD grown and ME MoS_2_ samples is even less than 1 when the adsorbates level is at the lowest vacuum level (1 × 10^−5^ mbar). This result shows that the PL intensity of CVD grown sample is comparable or even weaker than that of ME sample, which verifies that the room-temperature optical performance in vacuum could reflect the crystal quality. Figure 2d,e show the PL images of a typical CVD grown sample with grain boundary in air and vacuum, respectively. The sites at the grain boundary are expected with more defects and its decreased PL intensity measured in vacuum provides clear evidence that PL intensities at defect sites could be decreased when measured in vacuum.

Meanwhile, the *in-situ* Raman spectra have been obtained for these two groups of MoS_2_ samples, as shown in Fig. S6. There is no obvious change for the E^1^_2g_ mode (almost fixed at ~385.7 cm^−1^ for ME sample and ~384.4 for CVD grown sample), but a clear blue-shift of A_1g_ mode (from 404.94 cm^−1^ to 403.35 cm^−1^) for the CVD sample in vacuum compared with that in air, shown in Fig. S6a, which is larger than that of ME sample with a smaller shift from 404.21 cm^−1^ to 403.71 cm^−1^ (Fig. S6b). This result is different from the reported strain effect on the Raman spectra for monolayer MoS_2_ sample^28^, which could be further exclude the strain effect. The similar variation trends for A_1g_ and E^1^_2g_ mode have been reported by the gated Raman spectra from a 1 L MoS_2_^31^_._ The results show the softening of the A_1g_ mode with electron doping, while the frequency of E^1^_2g_ mode remains unchanged, which are due to the stronger electron-phonon coupling of the A_1g_ mode than that of the E^1^_2g_ mode^32,33^. So whether the shift of A_1g_ modeobserved in this work is due to the doping effect will be discussed later.

**Figure 3:**
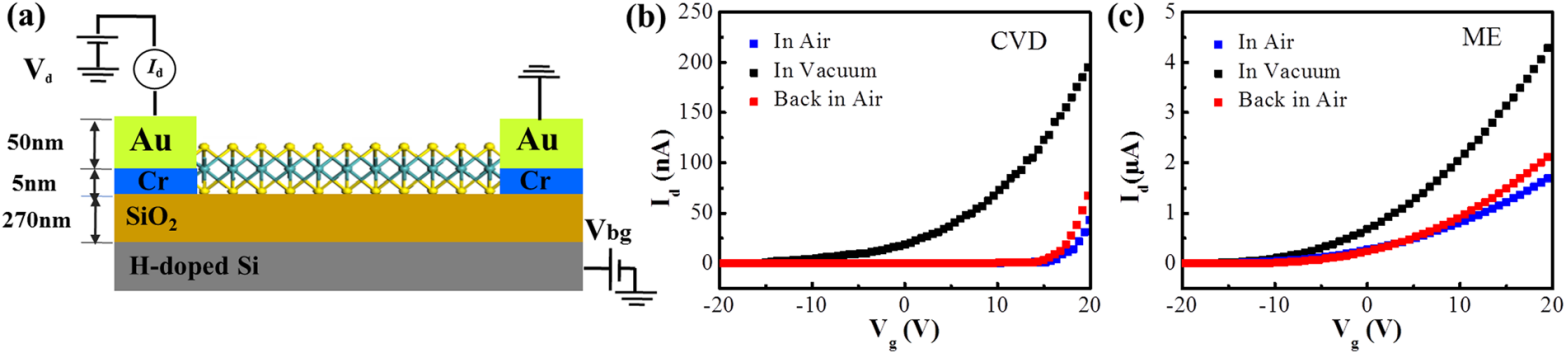
(**a**) The schematic of transistors used in this work. I_d_ -V_g_ curves of (**b**) CVD grown and (**c**) ME MoS_2_ transistors for V_bg_ = −20 V to 20 V measured in air, vacuum, and back to air, respectively. The fixed source-drain voltage is 1 V. The black, blue and red colour lines represent the curves measured in vacuum, air and back to air, respectively.

The vacuum-level dependent electrical performances of CVD grown and ME MoS_2_ are investigated. Figure 3a shows the schematic of a monolayer MoS_2_ based transistor fabricated in this work. The fabrication process is described in the experimental section. The optical images of FETs based on ME and CVD grown samples are shown in Fig. S7. Figure 3b,c show the electrical performance of single layer ME and CVD grown MoS_2_ based transistors, respectively. Both of these two groups transistors display n type behaviour, which mean excess electrons introduced in MoS_2_. Moreover, with the vacuum level decreasing, the threshold voltage shifts towards the positive direction, which means that the adsorbates in air play a p doping effect on MoS_2_ itself. Additionally, comparing the current at V_g_ = 0 V, the current is larger under vacuum when compared with that in air, which represents a stronger n doped effect in vacuum. While in air, due to the p doping effect from absorbates, the current is decreased. Both of CVD grown and ME samples show this variation trend. The mobility for CVD grown samples in vacuum is 0.69 cm^2^/VS while it is 44.2 cm^2^/VS for ME samples in vacuum, which means a poor crystal quality for CVD grown samples without doping effect from adsorbates. While this p-doping effect due to the adsorbates in air reduced the amounts of the excess electron in MoS_2_ and thus increased the radiative decay of exciton emission, which could be described by a three-level model including the excitation, exciton emission and trion emission processes^4,34^. As shown in Fig. S8a, G represents the generation rate of optical excitons. The radiative decay rates of the exciton and trion are marked as ${\Gamma }_{ex}$ and ${\Gamma }_{tr}$ respectively. When the mass action law is considered with trions together to evaluate the doped electron density in MoS_2_ samples, the integrated intensity ratio of trion emission (*I*_*tr*_) to electron emission (*I*_*ex*_) can be expressed as^34–37^:

1 $$\frac{{I}_{tr}}{{I}_{ex}}=\propto \frac{{n}_{e}}{{k}_{b}T}$$

*I*_*tr*_ and *I*_*ex*_ represent the trion and exciton emission intensities, respectively. *n*_*e*_ is the carrier concentration, *k*_*b*_ is the Boltzmann constant, and *T* represents temperature.

From equation (1), it is confirmed that the ratio of emission intensities from *I*_*tr*_ and *I*_*ex*_ indeed highly depends on the carrier concentration in MoS_2_, which corresponds to the experimental results shown in Fig. S8b. The fitting results for CVD grown and ME samples in air and vacuum are shown in Fig. S9, respectively, which directly reflect the vacuum level dependent intensity ratio of exciton and trion. Moreover, it is concluded that the binding energy of trion is ~24 meV. So their emission spectra overlapped with that of exciton emission since such negatively charged excitons usually have finite binding energies, ~20 meV^4^.

Moreover, such a p doing effect also explain well the Raman frequency shift of E^1^_2g_ and A_1g_ mode observed in Fig. S6. According to the symmetrical group theory, the A_1g_ mode has symmetrical lattice variation and would have a nonzero expectation value for the matrix element of the electron-phonon coupling, which leads to a larger electron-phonon coupling^31^. While for the E^1^_2g_ mode, the matric element of electron phonon coupling vanishes and its coupling with electrons is weaker on doping in ambient compared with A_1g_ modes^31–33^. Moreover, we have calculated the phonon energies of E^1^_2g_ mode and A_1g_ mode with and without considering the adsorbates, as shown in Fig. S10, the results are consistent to our experimental results and explanation above.

So far, the physical picture for the reason why the CVD grown sample shows a strong PL intensity is clear. The greater the amounts of adsorbates, the stronger the intensity of the PL emission in MoS_2_. Supposing there is no adsorption on the surface of MoS_2_, the PL intensity from CVD grown MoS_2_ should be weaker than that of ME sample due to its poorer crystal quality, which is corresponding to the experimental results on the intensity ratio (0.74) at higher vacuum shown in Fig. 2c.

**Figure 4:**
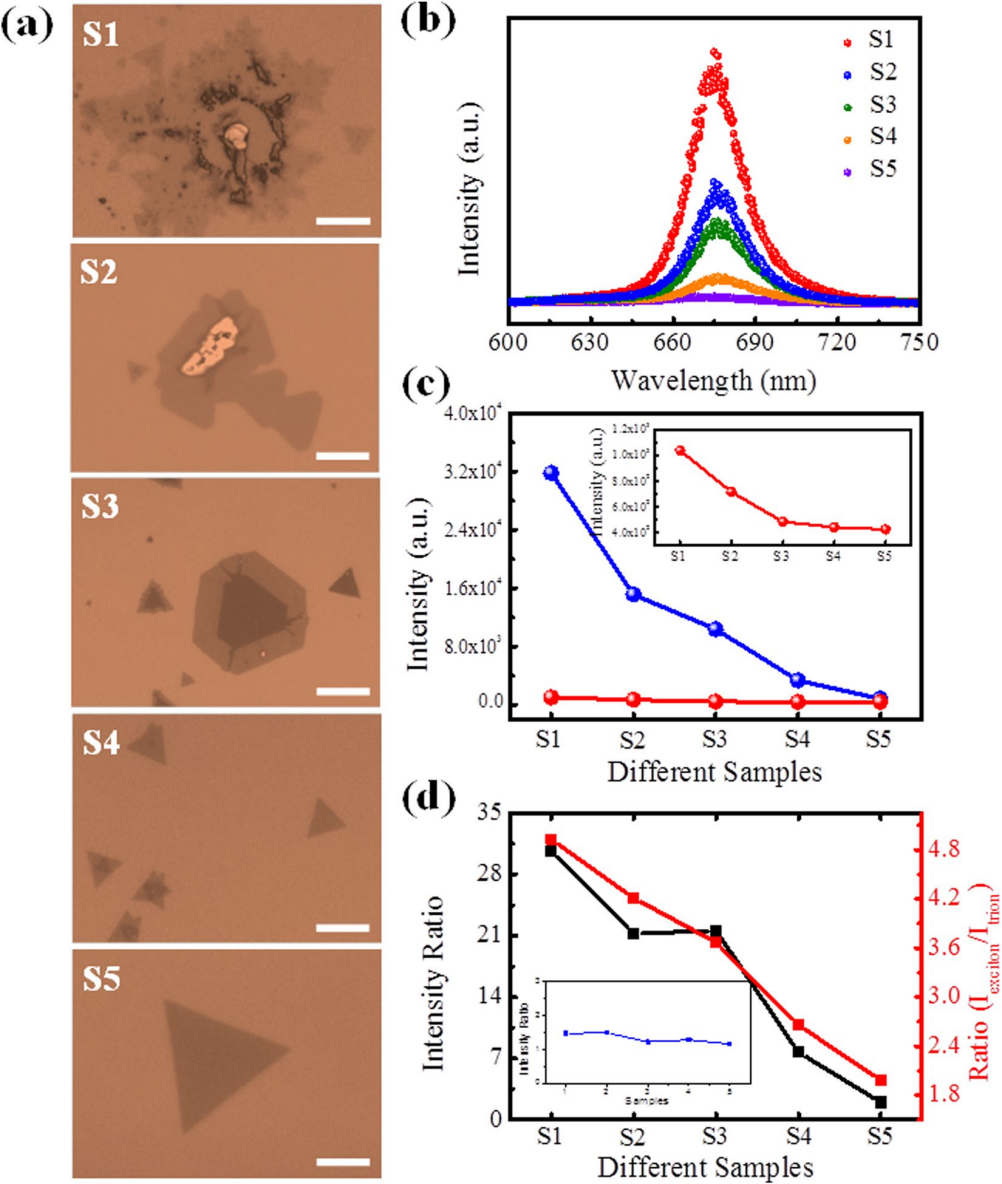
(**a**) Optical photos of CVD grown monolayer samples selected for PL measurement in (**b**), marked as S1, S2, S3, S4, S5. The scale bars shown in figures represent 15μm. (**b**) The corresponding PL spectra of CVD samples shown in (**a**). (**c**) The PL intensities of samples, shown in Figure (**b**), in air and in vacuum, respectively. The inset is the enlarged figure for the PL intensities in vacuum. (**d**) The ratios of PL intensities in air and vacuum for these five samples, and the intensity ratios of exciton and trion emissions for these five samples in air. The insert figure shows the intensity ratios of exciton and trion emission in vacuum.

Furthermore, the CVD grown monolayer MoS_2_ under different growth conditions marked as S1, S2, S3, S4, and S5, respectively, are investigated to confirm the vacuum level dependent PL spectra. Figure 4a shows the optical images of selected CVD samples. Their PL intensities are different in air (Fig. 4b), and their emission intensities are reduced measured in vacuum (1 × 10^−5^ mbar) by different extent, as shown in Fig. 4c. Figure 4d presents the ratios of PL intensities measured in air and vacuum for these samples. The different ratios when the vacuum level varies during measurement are due to their different amounts of adsorbates on the samples. The ratios of exciton and trion emissions for these five samples are also shown in Fig. 4d and the variation tread is almost the same as their vacuum-level dependent PL intensities. Additionally, compared with that in air, the ratios of exciton and trion emissions are decreased in vacuum which further confirms that the amounts of adsorbates are highly related to the PL intensity and the ratios of exciton and trion emission intensities in MoS_2_ and the optical performance of MoS_2_ in vacuum are positively related to its crystal quality.

To clarify the most likely components of adsorbates in air and how the adsorbates contribute to the p doping effect on MoS_2_, we have evaluated the charge transfer between adsorbates (O_2_, N_2_, OH^−^ and H_2_O molecules) and MoS_2_ by performing first-principle calculations as these three kinds of molecules are bountiful in air. We first consider the defects in MoS_2_. As shown in Fig. S11, we consider four typical types of defects by using a 4 × 4 supercell of MoS_2_ as the prototype: (1) a single S vacancy; (2) a single Mo vacancy; and (3) and (4) for two S vacancies at the same side and different sides. Our results show the single S vacancy is the most common defect with the lowest formation energies. Then we turn to focus on the charge transfer process between adsorbates and perfect (defective) MoS_2_. The binding energy (E_b_) of adsorbates A (O_2_, N_2_, OH^−^ and H_2_O) on pristine or defective monolayer MoS_2_ is given by

2 $${{\rm{E}}}_{{\rm{b}}}={\rm{E}}({{\rm{MoS}}}_{2}+{\rm{A}})-[{\rm{E}}({{\rm{MoS}}}_{{\rm{2}}})+{\rm{E}}({\rm{A}})]\,({\rm{A}}={{\rm{O}}}_{2},{{\rm{N}}}_{2},{{\rm{H}}}_{2}{\rm{O}})$$

**Figure 5:**
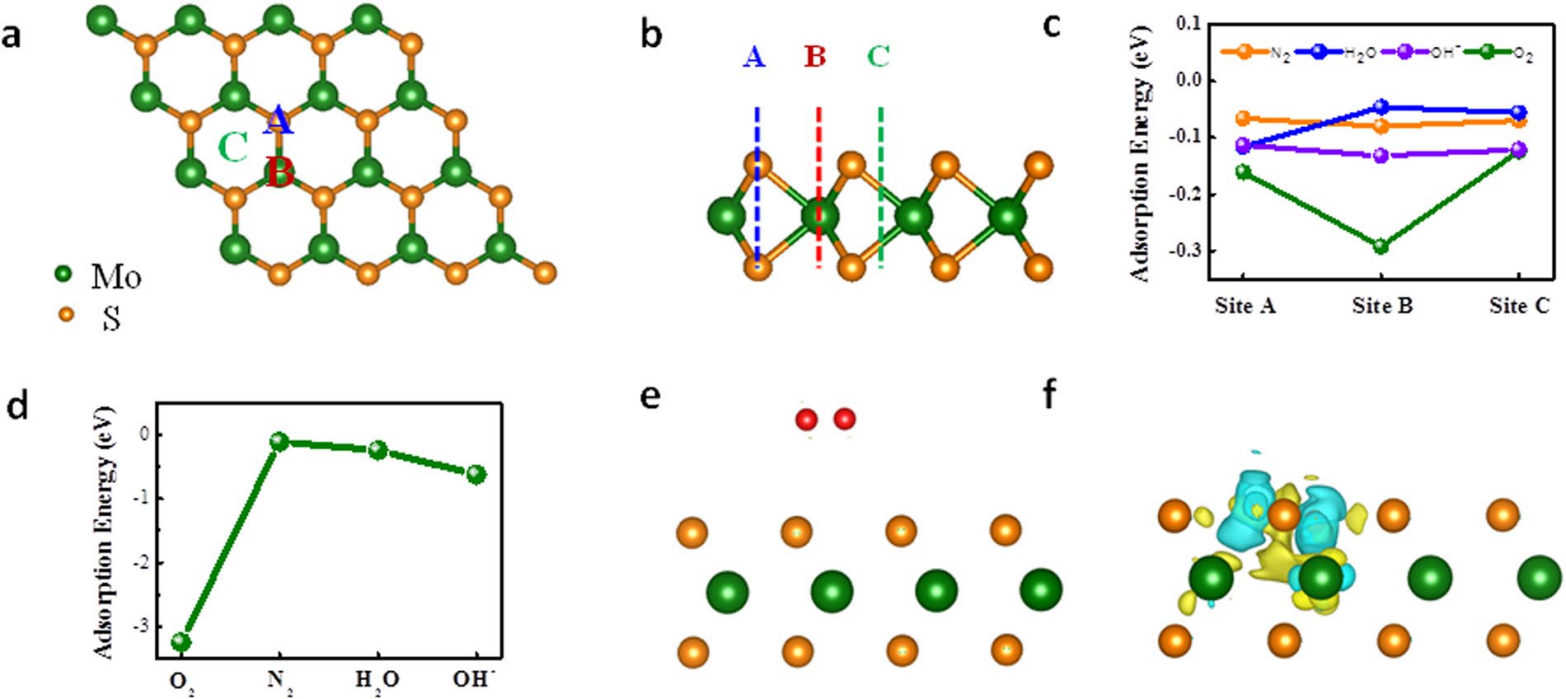
(**a**) Top and (**b**) side views of optimized structure of monolayer MoS_2_. A, B, C represent the different adsorption sites of adsorbates. (**c**) The adsorption energies of O_2_, N_2_, H_2_O and OH^−^ on A, B, C sites of pristine MoS_2_. (**d**) The adsorption energies of O_2_, N_2_, H_2_O and OH^−^ on defective MoS_2_ with a S vacancy. (**e**) and (**f**) Show the charge density difference of O_2_ molecule absorbed on pristine and defective 1 L MoS_2_ with a single S vacancy, respectively. The positive and negative charges are shown in yellow and blue colors, respectively. Isosurface values are 7.5 × 10^−2^ e/Å^3^ for (**e**) and 5 × 10^−3^ e/Å^3^ (**f**).

**Figure 6:**
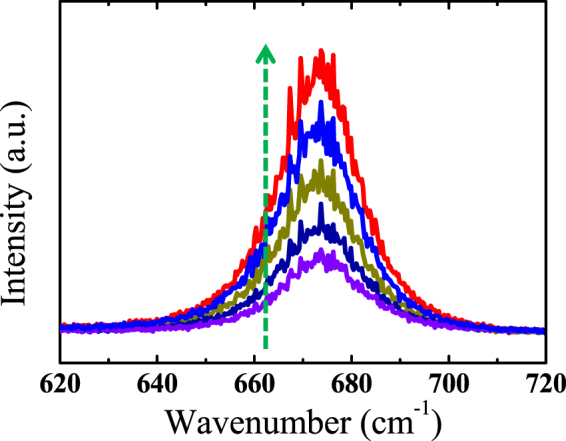
The PL spectra of annealed MoS_2_ under different oxygen atmosphere. The arrow represents the increase of oxygen amounts. The purple line represents the PL spectra of pristine sample.

All energies are calculated within the same supercell for comparison. For the pristine MoS_2_, three adsorption sites for O_2_, N_2_, OH^−^ and H_2_O molecules are schematically shown in Fig. 5a,b, and their binding energies with MoS_2_ are shown in Fig. 5c. The most favourable adsorption configurations for O_2_, N_2_ and OH^−^ are at B site, while for H_2_O it is at A cite, which are in good accordance with former calculations^38^. For the defective MoS_2_, we compare the adsorption energies of O_2_, N_2_, OH^−^ and H_2_O molecules on its surface. Figure 5d shows O_2_ molecule possesses the lowest adsorption energy (−3.25 eV), indicating O_2_ molecules are the most likely component to be adsorbed on defective MoS_2_. The O-H bond has a strong adsorption energy because it can form covalent bond on the Mo-edge^39^, but the sample used in this work is S terminated^40^. Therefore, we only discuss the adsorption of O_2_ molecules on the defect-sites of MoS_2_. Figure 5e,f show the amounts of charge transfer between perfect (defective) MoS_2_ and O_2_, which are 0.002e and 1.025e, respectively. Obviously, the charge transfers can be greatly enhanced with defects introduced in MoS_2_ and charges are extracted from defective MoS_2_ by O_2_ molecules due to its strong electronegativity. Thus, O_2_ molecules play a major role in the p-doping effect for MoS_2_. Moreover, to further confirm the doping effect of O_2_ molecules, we anneal the samples at 400 °C in one hour with different oxygen atmosphere (20, 30, 40, 50 Torr) and find the introduction of O_2_ indeed enhance the PL intensity of CVD grown monolayer MoS_2_ samples, as shown in Fig. 6. The simulated charge transfer process is highly consistent with the experimental results. The more defects in CVD samples which act as the localization centre could form the stronger localized exciton and increase the radiative recombination of exciton of MoS_2_.

## Conclusion

In summary, we clarify the origin of the abnormally strong PL in CVD grown samples and attribute it to the p doping effects of adsorbates at the defect-sites. HRTEM and EDS are used to confirm more defects in CVD grown samples than that in ME samples, and the *in-situ* vacuum level dependent PL and Raman spectra, as well as the electrical performance confirms our proposal. The CVD samples under different grown conditions are expected with different crystal qualities, which show similar variation trends by different extent which further confirm the observed phenomenon. First principle calculations were carried out to investigate the charge transfer process between MoS_2_ and adsorbates and clarify the p-doping effect of adsorbates due to the strong electronegativity of adsorbates. Such a p-doping effect from adsorbates reduces the concentration of excess electron in MoS_2_ and contributes to the radiative recombination process of exciton. This work proposes a new vacuum technique to evaluate the crystal quality and can guide the future works on the engineering of PL emission intensity on monolayer two-dimensional layered semiconductor materials.

## Methods

### Samples Preparation and Optical Characterization

Two groups of MoS_2_ samples were used in this experiment: (1) monolayer MoS_2_ exfoliated from nature MoS_2_ crystals (from SPI), and (2) CVD grown monolayer MoS_2_ (sulphur and MoO_3_ powders are placed in furnace, with a SiO_2_/Si substrate located face down above the MoO_3_ powder). The growth temperature is at 650 °C and this temperature is kept with 15 min. Raman and PL spectra were conducted on Witec CRM 300 confocal Raman microscopy. The excitation laser was 532 nm laser with a spot size about 500 nm. The output power measured from the objective lens was controlled below 0.5 mW to avoid damaging and heating on samples. The accumulation time for PL and Raman spectra was 10 s. The Raman spectra were collected by 1800 g/mm grating while PL spectra were collected by 300 g/mm grating.

The size of the probe X-ray beam is 200 μm. For sample viewing, firstly, an optical image is taken by an external camera. Based on that optical image, we zoom in and position to roughly the area that we want. Then, similar to a SEM/EDX, a finely focused x-ray beam is used to create a secondary electron image for sample viewing, and actual positioning for interested analysis area.

### Fabrication of Back-Gated MoS_2_ Transistors

Both of these two groups of MoS_2_ samples were deposited on 270 nm SiO_2_/Si substrates. The devices were fabricated by standard electron beam lithography, followed by the thermal evaporation of Cr/Au electrode, and lift-off process. The electric measurement system was carried out using Agilent B1500A semiconductor analyzer. The vacuum level is set at 1 × 10^−5^ Torr.

### First Principle Calculations

The first-principles calculations were realized based on the density functional theory (DFT), as implemented in the Vienna Ab initio Simulation Package (VASP)^41^. The exchange-correlation potential is chosen as generalized gradient approximation (GGA), formulated by Perdew-Burke-Ernzerhof (PBE) functional^42,43^. The cut off energy of 400 eV is used for the plane-wave expansion of valence electron-wave functions. To avoid artificial interaction between layers, a vacuum spacing of >15 Å is built. In the calculations, DFT-D2 method is employed to describe the long-range van der Waals interactions^44^. For both defects identifications and gas molecules adsorptions on the monolayer MoS_2_, the calculations were performed on a 4 × 4 × 1 size of MoS_2_ supercell, with Monkhorst-Pack k-point meshes of 7 × 7 × 1^45^. The convergence criteria for energy and force are set to be 10^−5^ eV and 0.01 eV Å^−1^, respectively. The amount of charge transfers between gas molecules and MoS_2_ monolayer is estimated by using the Bader charge method.

## Electronic supplementary material

Supporting information
