# Supplementary material for: Vacuum level dependent photoluminescence in chemical vapor deposition-grown monolayer MoS2
Source: Sci Rep. 2017 Dec 1;7:16714. doi: 10.1038/s41598-017-15577-1 (PMC5711928; doi:10.1038/s41598-017-15577-1)
Supplement: Supplementary file 1 — Supporting information [file 41598_2017_15577_MOESM1_ESM.pdf]

Supporting Information

Vacuum level dependent photoluminescence in chemical vapor deposition-grown monolayer MoS_2

Linfeng Sun,[1,2] Xiaoming Zhang,[1] Fucai Liu,[3] Youde Shen,[4] Xiaofeng Fan,[5] Shoujun Zheng,[2] John TL Thong,[4] Zheng Liu,[3,6] Shengyuan A. Yang,[1] and Hui Ying Yang*,[1]

[1] Pillar of Engineering Product Development, Singapore University of Technology and Design, Singapore 487372, Singapore, E-mail: yanghuiying@sutd.edu.sg
[2] Division of Physics and Applied Physics, School of Physical and Mathematical Science, Nanyang Technological University, Singapore 637371, Singapore
[3] Center for Programmable Materials, School of Materials Science and Engineering, Nanyang Technological University, Singapore 639798, Singapore
[4] Department of Electrical and Computer Engineering, National University of Singapore, Singapore 117583, Singapore
[5] College of Materials Science and Engineering, Jilin University, Changchun 130012, P. R. China
[6] Centre for Micro-/Nano-electronics (NOVITAS), School of Electrical & Electronic Engineering, Nanyang Technological University, Singapore 639798, Singapore

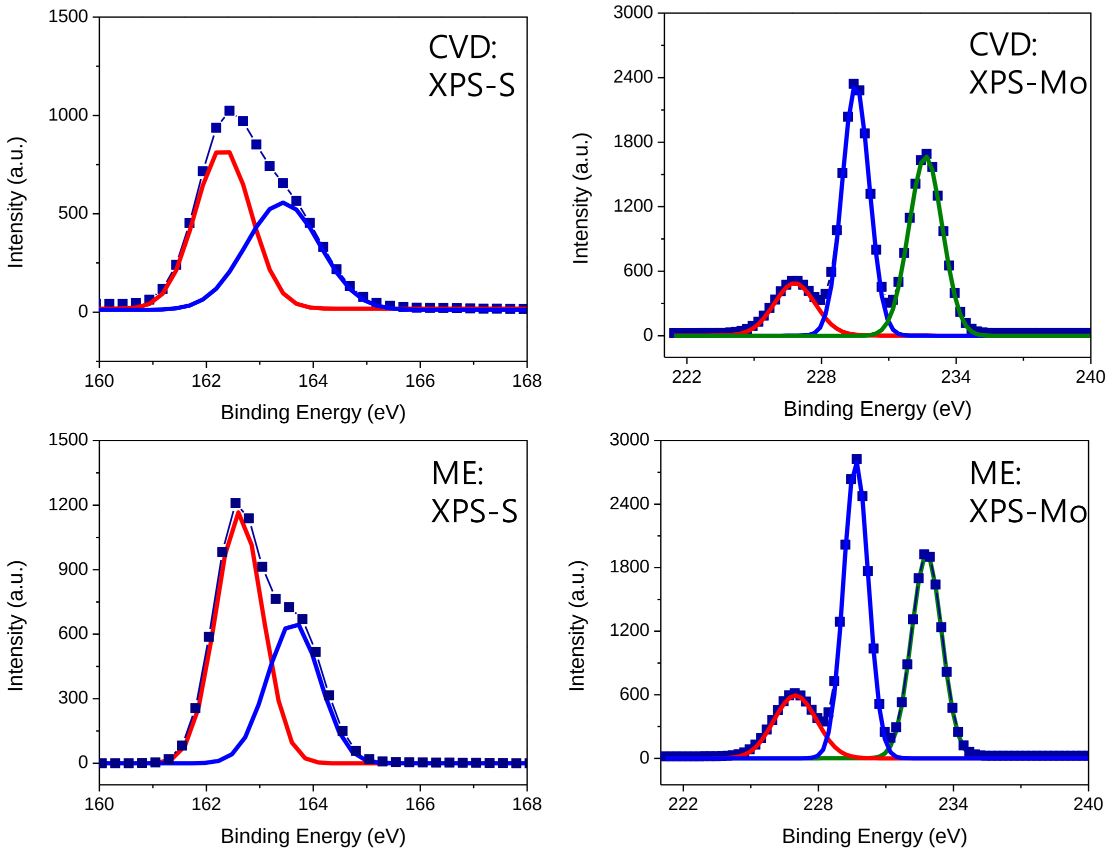

Figure S1. XPS spectra of Mo and S for CVD grown and ME monolayer MoS_2, respectively. The blacks line with square symbols are the experimental data and the continuous lines are fitted curves.

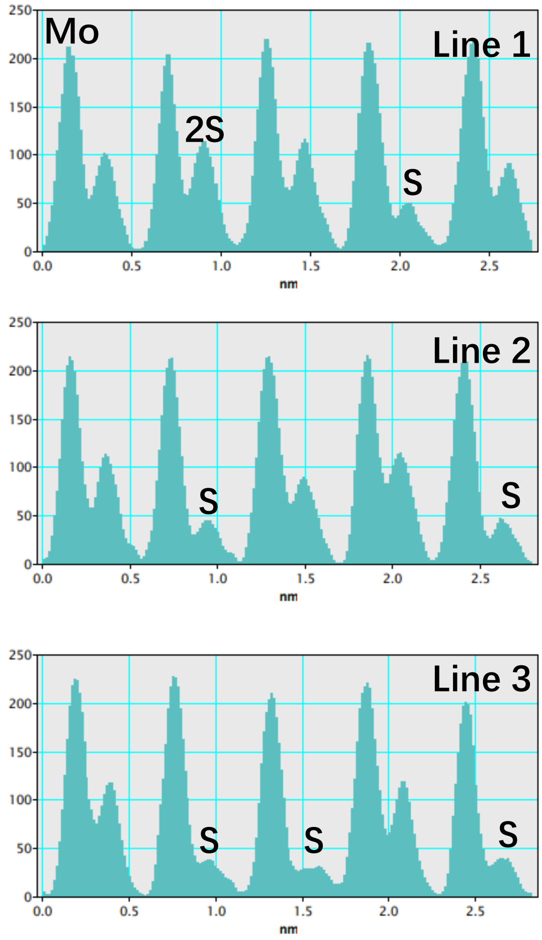

Figure S2. Intensity line profiles measured in the Figure 1c.

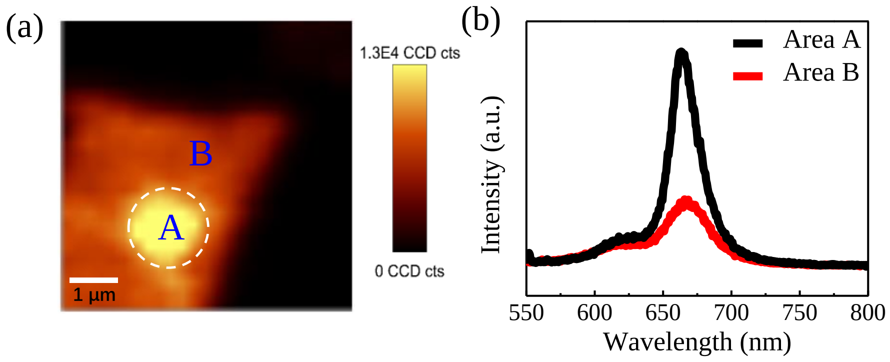

Figure S3. (a) PL mapping of monolayer MoS_2, which is normalized to the intensity of A exciton. The bright spot is pre-treated by argon plasma with a TEM grid as mask. (b) PL spectra of 1L MoS_2 are measured from area A and B, respectively.

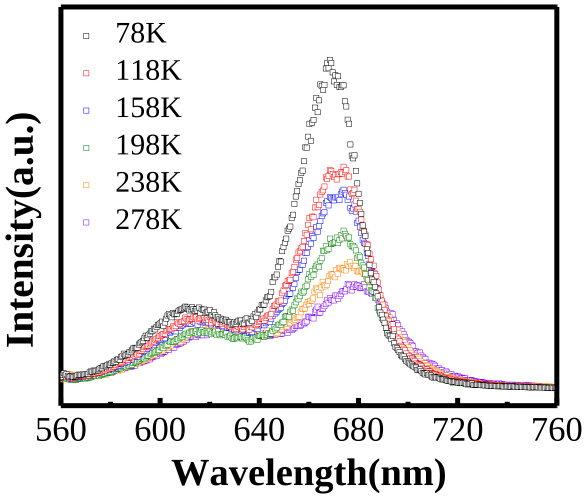

Figure S4: Temperature dependent PL spectra of monolayer MoS_2. The experiments were carried out by LINKAM stage with sample in it.

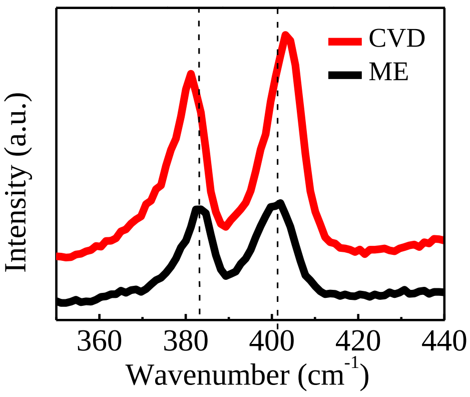

Figure S5. Raman spectra of CVD grown and ME monolayer MoS_2 samples.

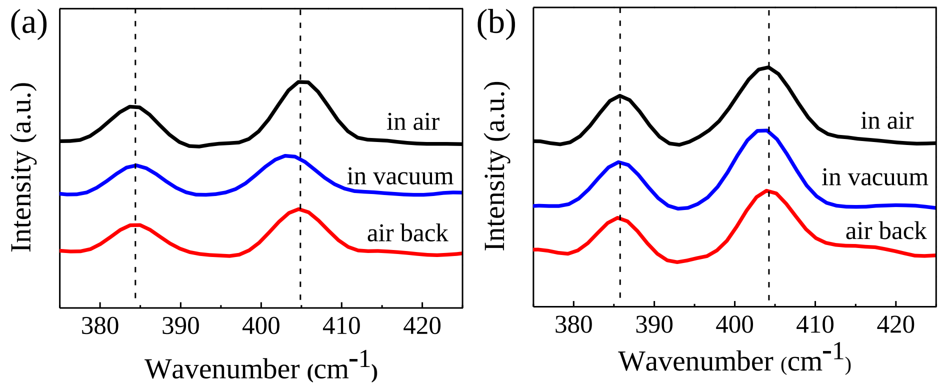

Figure S6. a,b) The in-situ Raman spectra of CVD and ME monolayer MoS_2 measured in air, vacuum, and in ari again.

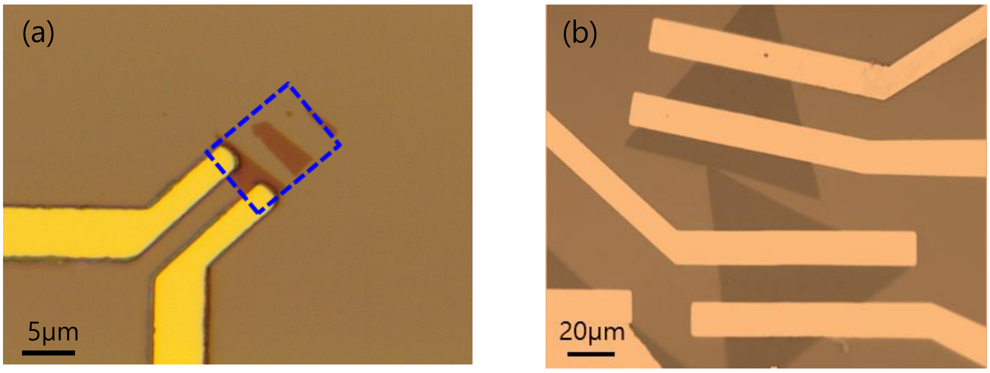

Figure S7: The optical image of FETs based on ME and CVD grown monolayer MoS_2 sample, respectively.

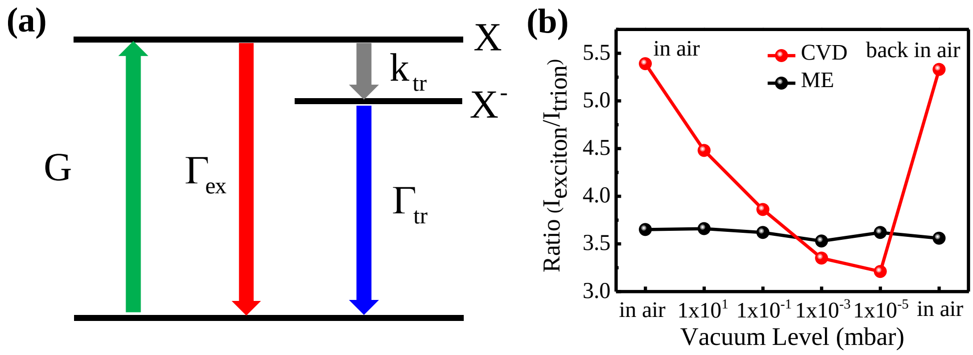

Figure S8: (a) Schematic of three-level energy diagram. The exciton and trion emission processes are denoted as Γ_{ex} and Γ_{tr}, respectively. G represents the photoexcitation process and the k_{tr} represents the formation of trion. (b) Vacuum level dependent intensity ratios of exciton to trion emissions in CVD grown and ME samples, respectively.

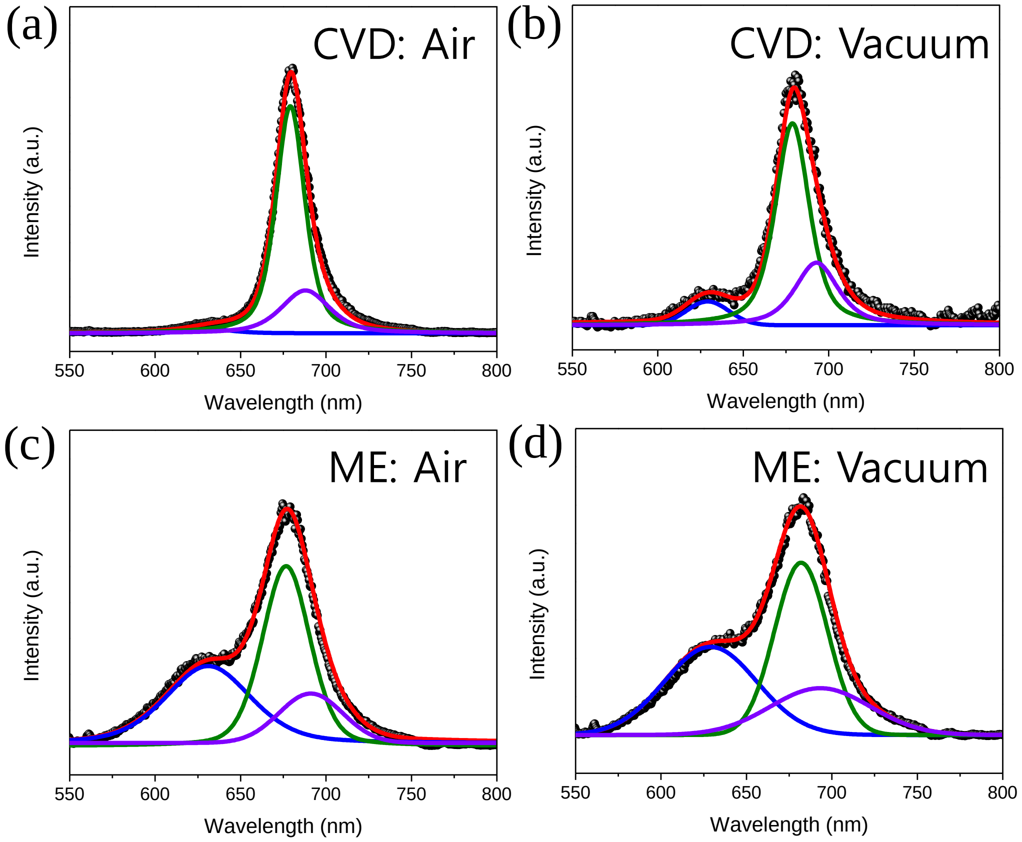

Figure. S9: The fitting spectra of CVD grown and ME MoS_2 samples in air and the highest vacuum level in this work. The vacuum level dependent emission ratio of exciton/trion is shown.

S10: Phonon energies with and without considering the adsorbates

Cases	E^1_{2g} (cm^{-1})	A_{1g} (cm^{-1})
**Without adsorbates**	**378.58**	**401.4**
**With adsorbates**	**377.24**	**403.61**

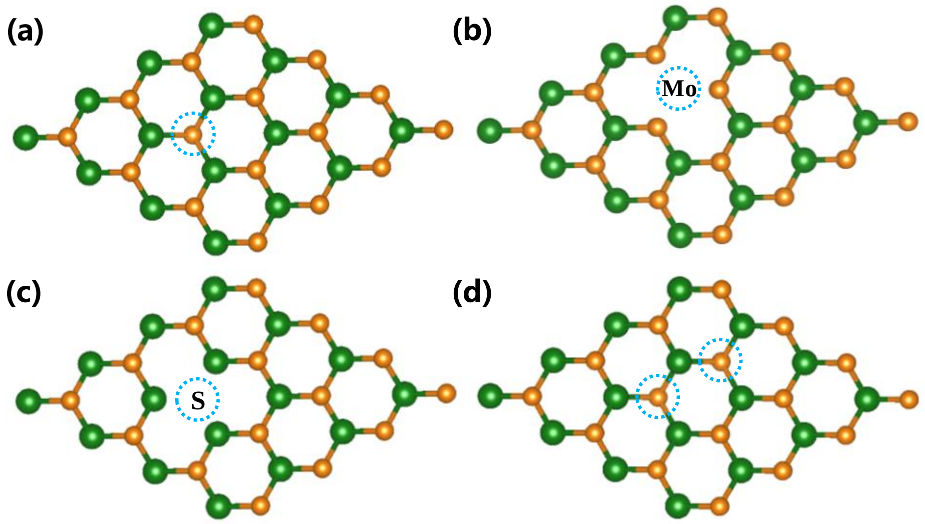

Figures S11: Possible point defects in 1L MoS_2. (a) single S vacancy; (b) double S vacancies at different sides; (c) two S vacancies at the same side; (d) single Mo vacancy. The formation energies (ΔE_{Form}) are calculated to be 0.96 eV, 1.88 eV, 1.82 eV, 4.53 eV, respectively.

$\Delta E_{Form} = E_{no\text{-}defect} - E_{defect} - N_S \times E_{S\_1L} - N_{Mo} \times E_{Mo\_1L}$

$E_{S\_1L} = E_{S(single)} + E_{Bond}$

$E_{Mo\_1L} = E_{Mo(single)} + 2E_{Bond}$

$E_{Bond} = (E_{MoS2\_1L} - E_{Mo(single)} - 2E_{S(single)})/3$

Where $E_{no\text{-}defect}$ and E_{defect} are the total energies of pristine and defective MoS_2 supercell, E_{S_1L} and E_{Mo_1L} are the energies of single Mo and S in a perfect monolayer, and E_{Bond} is the strength of Mo-S bond in MoS_2.
